# Cerebral Phosphorus Magnetic Resonance Spectroscopy in a Patient with Giant Cell Arteritis and Endovascular Therapy

**DOI:** 10.1155/2018/7806395

**Published:** 2018-10-28

**Authors:** Ruth Steiger, Lisa-Maria Walchhofer, Andreas Rietzler, Katherina J. Mair, Michael Knoflach, Bernhard Glodny, Elke R. Gizewski, Astrid E. Grams

**Affiliations:** ^1^Department of Neuroradiology, Medical University of Innsbruck, Anichstraße 35, 6020 Innsbruck, Austria; ^2^Department of Radiology, Medical University of Innsbruck, Innsbruck, Austria; ^3^Department of Neurology, Medical University of Innsbruck, Innsbruck, Austria

## Abstract

With phosphorus magnetic resonance spectroscopy (31P MRS) energy metabolites can be visualised. In this case study, we report on a patient with stenosis and wall contrast enhancement in the left internal carotid and the right vertebral artery, due to giant cell arteritis. 31P MRS revealed a decreased inorganic phosphate-to-phosphocreatine ratio (Pi/PCr) in regions with a prolonged mean transit time (MTT). After systemic therapy and angioplasty of the right vertebral artery, the stenosis and the symptoms improved and the area of prolonged MTT became smaller. However, a new decrease in Pi/PCr in areas that developed moderately prolonged MTT was observed.

## 1. Introduction

Giant cell arteritis (GCA) is a common form of primary arteritis in patients over 50 years, in which supraaortal arteries are predominantly affected [1, 2]. Typical symptoms include headache, jaw claudication, scalp tenderness, pain in the temple, and sudden loss of vision. In rare cases symptoms are night-sweating or weight loss [3]. For the diagnosis of GCA, three out of the five following criteria must be met: age over 50 years, new onset of localised headache, raised erythrocyte sedimentation rate (ESR) of > 50 mm/1st hour, tenderness, nodulesm or reduced pulse of the temporal arterym or abnormal arterial biopsy findings [4]. Brain ischaemia resulting from GCA affecting also intracranial vessels has been reported in 3-4% of the patients. Inflamed vessel segments can be identified by magnetic resonance imaging (MRI) [5], cerebral infarcts can be visualised with diffusion weighted imaging, and brain ischaemia can be estimated by perfusion weighted imaging (PWI), for instance, by measuring a prolongation of the mean transit time (MTT) [6]. First line therapy is drug related to steroids or other immunosuppressant medication. Revascularisation of the affected vessels can be achieved by angioplasty or stent implantation in selected patients, as the endovascular procedure can be high risk [2, 7].

**Figure 1:**
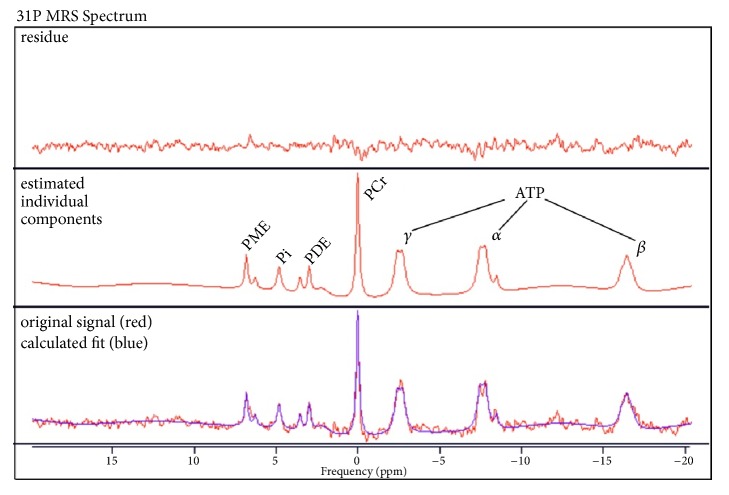
Examples for phosphorous magnetic resonance spectrum. The peaks of the different detectable membrane and energy metabolites from left to right: phosphomonoesters (PME), inorganic phosphate (Pi), phosphodiesters (PDE), and phosphocreatine (PCr), as well as three resonances of adenosine triphosphate (gamma-, alpha-, and beta-ATP). The vertical axis displays arbitrary units, while the x-axis shows the chemical shift difference to PCr in ppm. The original signal is depicted as the red curve and the calculated fit can be seen in blue.

Phosphorus magnetic resonance spectroscopy (31P MRS) enables the assessment of different energy and membrane metabolites in vivo. The number of metabolites corresponds to the area under the fitted curve of the respective peak (Figure 1). Prior studies reported changes in phosphocreatine (PCr), adenosine triphosphate (ATP), and inorganic phosphate (Pi) in patients with high-grade carotid artery stenosis [8]. However, in order to receive comparable results, it is common to interpret MRS data as ratios between different metabolites [9].

We present the 31P MRS findings in relation to MR perfusion imaging in a patient with severe brain ischaemia and intermittent transient ischaemic attacks due to giant cell arteritis prior to and after systemic and endovascular therapy.

## 2. Case Report

A 67-year-old Caucasian male presented with recurrent episodes of amaurosis fugax, sharp pain in both temples, masticatory claudication, intermitting paresis of the right arm, and a positive right-sided Babinski sign. ESR was 93 mm/1st hour, C-reactive protein was elevated up to 14.18 mg/dl, and fibrinogen was 1062 mg/dl. The diagnosis of giant cell arteritis was established. Additionally, the patient suffered from arterial hypertension, type 2 diabetes mellitus, hypercholesterinaemia, coronary heart disease, and paroxysmal atrial fibrillation.

**Figure 2:**
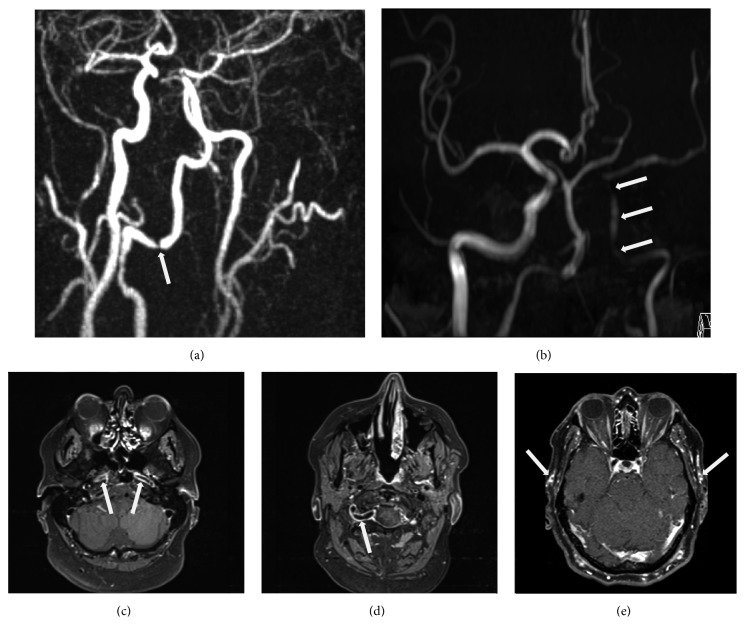
Magnetic resonance images of the affected arteries. High-grade stenosis of the right vertebral and the left distal internal carotid artery ((a), arrow) on a contrast enhanced magnetic resonance angiography. Long stenosis of the left ICA with tapering aspect ((b), arrows) on a follow-up time of flight MRI. Vessel wall enhancement of both internal carotid arteries ((c), arrows), and the right vertebral artery ((d), arrow), visible on a “dark blood” sequence. Contrast enhancement of both superficial temporal arteries ((e), arrows).

The patient received a structural MRI scan with a 3T whole-body system (Verio, Siemens Medical 22 AG, Erlangen, Germany) and a 12-channel reception head coil. MRI angiography revealed short high-grade stenosis of the right vertebral artery (VA) in the V3 segment (Figure 2(a)), a hypoplastic left VA, and a patent posterior communicating artery on the right side. The C6 and C7 segments of the left internal carotid artery (ICA) also showed high-grade stenosis (Figure 2(a)). On a follow-up MRA three weeks later especially the stenosis in the left ICA was longer, but also the stenosis of the right VA (Figure 2(b)). The walls of both ICA (Figure 2(c)), the left temporal artery (TA), and the right VA (Figure 2(d)) were thickened with contrast enhancement, so were the walls of the superficial temporal arteries (Figure 2(e)). In addition, a left-sided pontine infarct was present. Proton emission tomography computed tomography (PET-CT) found no involvement of other noncranial vessels. The diagnosis was based on the 1990 ACR criteria, in which the presence of three out of five points results in a sensitivity of 93.5 % and a specificity of 91.9 % [10]. Even though a halo sign was not seen in color Doppler ultrasound we did not perform a temporal artery biopsy due to the vascular high-risk situation with the need of pronounced antithrombotic therapy. When we retrospectively applied the revised 2016 criteria of the ACR (Sait et al. 2017) for the diagnosis of GCA, we would still confirm the diagnosis with at least four points (three of those in Domain I).

The patient was treated with high-dose corticosteroids as well as acetylsalicylic acid. Within two days, his symptoms had resolved. ESR slowed down to 23 mm/1st hour.

**Figure 3:**
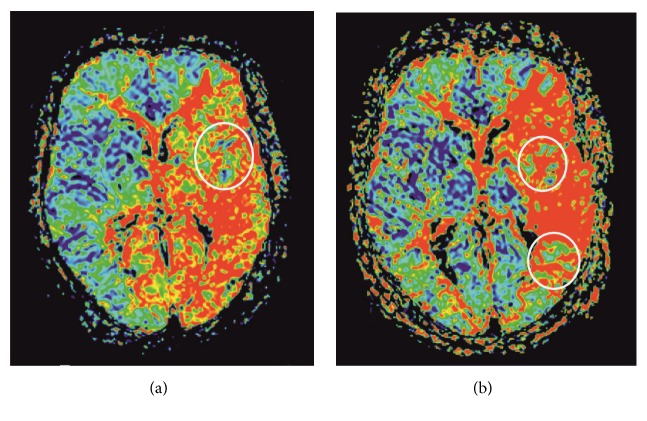
Perfusion weighted imaging prior to and after therapy. Prior to therapy (a): prolonged mean transit time in large parts of the left middle cerebral artery territory as well as both posterior cerebral artery territories and an area with only moderately elevated transit time was found in the left insular cortex (white circle). After endovascular therapy (b): improvement of cerebral perfusion in both posterior cerebral artery territories and partly the left middle cerebral artery territory, with a now moderately elevated transit time in the border zone between middle and posterior cerebral artery territories, in addition to the area in the insular cortex (circles).

16 days after his first presentation, the patient suffered a new onset of acute aphasia and right-sided facial palsy. In the following days, neurological symptoms fluctuated from mild aphasia to severe aphasia, which could not be stabilised by moderate hypertension, dual antiplatelets, or anticoagulation. A brain MRI revealed a new infarct in the left basal ganglia and the left centrum semiovale. The left ICA stenosis had become more pronounced and extensive compared to the initial MRI, with the C5 segment now involved as well. PWI showed that the MTT, but none of the other perfusion parameters, was inhomogeneously prolonged in the left middle cerebral artery (MCA) territory and in both posterior cerebral artery (PCA) territories (see Figure 3(a)). Additionally to these sequences, a 31P MRS sequence was acquired, with an acquisition time of 10:44, a repetition time of 2000 ms and an echo time of 2.3 ms. The volume of interest was gained with an extrapolated 16 x 16 x 8 matrix and a field of view of 240 x 240 x 200 mm^3^, resulting in a voxel size of 15 x 15 x 25 mm^3^. For its acquisition the patient had to sit up briefly and the head coil was changed to a double-tuned 1H/31P volume head coil (Rapid 23 Biomedical, Würzburg, Germany). 31P-MRS data was postprocessed offline with the software package jMRUI version 5.0 (current stable version 5.4 available at http://www.jmrui.eu/), utilizing prior knowledge for the nonlinear least square fitting algorithm AMARES [11]. The fitting model was composed of 15 Lorentzian-shaped exponentially decaying sinusoids; however, for this patient only the calculation of the metabolite ratio of Pi/PCr was taken into account, as this ratio can be seen as a marker for the energy reserve [12]. 31P MRS revealed a decreased Pi/PCr ratio (Table 1) in both PCA and central left MCA territories in areas which showed a moderately prolonged (3.432 sec) but shorter MTT (Figure 3(a), Table 1) than surrounding areas (3.776 sec, reference value contralateral MCA territory = 3.279 sec). However the adjacent area showed a higher Pi/PCr ratio than the contralateral MCA territory (Table 1).

**Figure 4:**
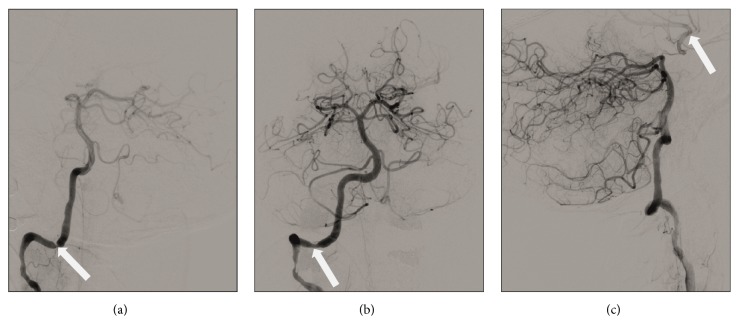
Conventional angiography prior to and after angioplasty. Contrast injections in the right vertebral artery, displaying a high-grade stenosis of the right vertebral artery ((a), arrow), which improved after stent angioplasty ((b), arrow). After angioplasty a new cross-flow via the left posterior communicating artery to the left distal internal carotid artery is seen ((c), arrow).

The therapeutic consequences were an increase of the corticosteroid dosage and initiation of an interleukin-6 receptor blocker therapy. Due to the fluctuating neurological symptoms—with large mismatches between areas with prolonged MTT and the clinical presentation—various potential interventional strategies were discussed. Angioplasty of the left ICA was deemed to be higher risk, because the affected intradural segments were very elongated. With the intention to improve the perfusion of the ACM territory via the posterior communicating artery, the high-grade stenosis in the V3 segment of the right vertebral artery was corrected via balloon angioplasty and stenting (Figure 4). The intervention was successful, and the aphasia improved rapidly. Follow-up MRI revealed an improved perfusion in parts of the left MCA and both PCA territories. The Pi/PCr ratio in the MCA territory was higher than in the first scan and higher than in the contralateral hemisphere (Table 1). 31P MRS showed a newly decreased Pi/PCr ratio in the border area between MCA and PCA territories and the insular cortex of the left side (Figure 3(b); Table 1), again in an area with a moderately increased MTT (3.462 sec), which was shorter than in the ventral adjacent MCA territory (3.962 sec; Figure 3(b)).

After two months, all mentioned stenoses improved and further clinical improvement was observed. Only a slight aphasia persisted. The corticosteroid dose was able to be reduced to a maintenance level.

## 3. Discussion

In this case, energy metabolism changes in a patient suffering from cerebral ischaemia due to giant cell arteritis could be visualised with 31P MRS, both before and after stent angioplasty of the right VA. Prior to the intervention, 31P MRS showed a decreased Pi/PCr ratio in both PCA territories and the central left MCA territory, in areas with a moderately prolonged MTT. In unaffected areas and those areas with a more severely prolonged MTT, no changes of the energy metabolites were found.

Former studies have reported the impact of carotid stenosis or ischaemic stroke on brain energy metabolism [8, 13]. In patients with high-grade carotid artery stenosis, decreased concentrations of ATP and Pi were observed in comparison to healthy controls, and decreased PCr was found in untreated patients [8]. Our findings of a decreased Pi/PCr ratio in less perfused brain tissue are consistent with these results. After VA angioplasty, MTT improved in the left MCA and both PCA territories. However, 31P MRS revealed a newly decreased Pi/PCr ratio in two additional areas with now moderately prolonged MTT. Similar to a prior study [8], we also found a generalised decrease of Pi/PCr in both hemispheres after endovascular therapy.

This is the first time that cerebral 31P MRS has been described to evaluate the therapeutic effects of intracranial angioplasty in giant cell arteritis. Our findings suggest that the fast energy metabolism pathway—the creatine kinase reaction—is a dynamic process, which seems to take place only in areas with moderately but not severely prolonged blood transition. This energy provision seems to be able to adapt according to perfusion circumstances. Our findings imply that the fast energy supply might only take place in less affected areas, which are more likely to survive an ischaemic situation. This in vivo insight into brain energy metabolism might be helpful in selecting patients for interventional treatment of cerebral vessel stenosis in the future. A combination of PWI and 31P MRS could be a novel supplementary method to penumbra imaging, helping to identify tissues at risk of infarction more accurately, for example, in patients with “wake-up” strokes. This could provide more safety for the indication of thrombectomy, intracranial stenting, or bypassing in different situations or display a tool for therapy monitoring. However, the clinical application of this multimodal MRI method has to be evaluated in prospective studies and in a larger sample size of patients with stenosis of the supraaortal or intracranial arteries.

A major limitation of the 31P MRS method is that it is only available in a few centres that the acquisition time is quite long (11 mins) and that the voxel size is larger than most of the regions of interest (15 x 15 x 25 mm). Further modifications with a faster acquisition method and a better spatial resolution could lead to a wider applicability of this method.

## 4. Conclusion

31P MRS, in combination with the established PWI, is a promising method to evaluate the complex processes that occur during cerebral infarct or ischaemia. Not only does it provide useful information about the perfusion of small vessels, it also gives insight into energy metabolism on a cellular level. Further studies on a larger scale are highly recommended.

## Figures and Tables

**Table 1:** Results of mean transit time (MTT, given in seconds) and inorganic phosphate to phosphocreatine ratio (Pi/PCr, given in arbitrary units).

**Date of exam**	**Area**	**MTT**	**Pi/PCr ** **Mean value**
02.07.2015	central MCA	3.43	3.28

02.07.2015	adjacent	3.78	5.49

02.07.2015	contralateral	3.28	4.47

23.07.2015	central MCA	3.96	5.79

23.07.2015	MCA/PCA border	3.46	3.16
